# Impact of red blood cell transfusion on oxygen transport and metabolism in patients with sepsis and septic shock: a systematic review and meta-analysis

**DOI:** 10.5935/0103-507X.20210017

**Published:** 2021

**Authors:** María Camila Arango-Granados, Mauricio Umaña, Álvaro Ignacio Sánchez, Alberto Federico García, Marcela Granados, Gustavo Adolfo Ospina-Tascón

**Affiliations:** 1 Fundación Valle del Lili - Cali, Valle del Cauca, Colombia; 2 Universidad ICESI - Cali, Valle del Cauca, Colombia.

**Keywords:** Sepsis, Shock, septic, Erythrocyte transfusion, Oxygenation, Oxygen consumption, Microcirculation, Spectroscopy, near-infrared, Sepse, Choque séptico, Transfusão de eritrócitos, Oxigenação, Consumo de oxigênio, Microcirculação, Espectroscopia de luz próxima ao infravermelho

## Abstract

Red blood cell transfusion is thought to improve cell respiration during septic shock. Nevertheless, its acute impact on oxygen transport and metabolism in this condition remains highly debatable. The objective of this study was to evaluate the impact of red blood cell transfusion on microcirculation and oxygen metabolism in patients with sepsis and septic shock. We conducted a search in the MEDLINE^®^, Elsevier and Scopus databases. We included studies conducted in adult humans with sepsis and septic shock. A systematic review and meta-analysis were performed using the DerSimonian and Laird random-effects model. A p value < 0.05 was considered significant. Nineteen manuscripts with 428 patients were included in the analysis. Red blood cell transfusions were associated with an increase in the pooled mean venous oxygen saturation of 3.7% (p < 0.001), a decrease in oxygen extraction ratio of -6.98 (p < 0.001) and had no significant effect on the cardiac index (0.02L/minute; p = 0,96). Similar results were obtained in studies including simultaneous measurements of venous oxygen saturation, oxygen extraction ratio, and cardiac index. Red blood cell transfusions led to a significant increase in the proportion of perfused small vessels (2.85%; p = 0.553), while tissue oxygenation parameters revealed a significant increase in the tissue hemoglobin index (1.66; p = 0.018). Individual studies reported significant improvements in tissue oxygenation and sublingual microcirculatory parameters in patients with deranged microcirculation at baseline. Red blood cell transfusions seemed to improve systemic oxygen metabolism with apparent independence from cardiac index variations. Some beneficial effects have been observed for tissue oxygenation and microcirculation parameters, particularly in patients with more severe alterations at baseline. More studies are necessary to evaluate their clinical impact and to individualize transfusion decisions.

## Abstract (pt)

Considera-se que a transfusão de eritrócitos melhora a respiração celular durante o choque séptico. Contudo, seu impacto agudo no transporte e no metabolismo de oxigênio nessa condição ainda é amplamente debatido. O objetivo deste estudo foi avaliar o impacto da transfusão de eritrócitos na microcirculação e no metabolismo do oxigênio em pacientes com sepse e choque séptico. Conduzimos um levantamento nas bases de dados MEDLINE^®^, Elsevier e Scopus. Incluímos estudos realizados com seres humanos adultos com sepse e choque séptico. Realizamos uma revisão sistemática e metanálise com utilização do modelo de efeitos aleatórios de DerSimonian e Laird. Consideramos significante valor de p < 0,05. Incluíram-se na análise 19 manuscritos, correspondentes a 428 pacientes. As transfusões de eritrócitos se associaram com aumento de 3,7% na média combinada de saturação venosa mista de oxigênio (p < 0,001), diminuição de razão de extração de oxigênio de -6,98 (p < 0,001) e nenhum efeito significante no índice cardíaco (0,02 L/minuto; p = 0,96). Obtiveram-se resultados similares em estudos que incluíram mensurações simultâneas de saturação venosa mista de oxigênio, razão de extração de oxigênio e índice cardíaco. As transfusões de eritrócitos levaram a aumento significante na proporção de pequenos vasos perfundidos (2,85%; p = 0,553), enquanto os parâmetros de oxigenação tissular revelaram aumento significante no índice de hemoglobina tissular (1,66; p = 0,018). Estudos individuais relataram melhoras significantes na oxigenação tissular e nos parâmetros microcirculatórios sublinguais em pacientes com microcirculação alterada na avaliação inicial. A transfusão de eritrócitos pareceu melhorar o metabolismo sistêmico de oxigênio com aparente independência de variações no débito cardíaco. Observaram-se alguns efeitos benéficos para a oxigenação tissular e parâmetros microcirculatórios, em particular em pacientes com alterações iniciais mais graves. São necessários mais estudos para avaliar seu impacto clínico e individualizar as decisões relativas à transfusão.

## INTRODUCTION

Anemia is a common condition in critically ill patients.^(1)^ Multiple mechanisms have been implicated in its development, many of which are unrelated to active bleeding.^(1-4)^ Low hemoglobin (Hb) levels are generally related to increased morbidity^(1)^ and mortality,^(2)^ probably as a consequence of the metabolic imbalance between oxygen demands and supply and the sustained hyperadrenergic compensatory response. This is why allogeneic red blood cell (RBC) transfusions are used to treat moderate anemia, aiming to increase the oxygen-carrying capacity to the tissues and thus to meet cellular metabolic demands. However, some evidence suggests that RBC transfusion can be harmful,^(3)^ while conservative transfusion strategies lead to similar or even better clinical outcomes than liberal transfusion practices,^(5-10)^ thus suggesting that moderate or even low Hb levels are apparently well tolerated in humans without significant comorbidities and during more stable phases of disease.

Red blood cell transfusions might increase the risk of harm in patients with normal oxygen metabolism.^(11-13)^ Thus, guiding transfusions by metabolic or physiological variables would be more plausible than transfusing RBCs and aiming at a predetermined Hb level. However, Hb levels are habitually used as a trigger and target for RBC transfusions in clinical practice,^(5-10)^ assuming that maintaining Hb levels should ensure adequate oxygen transport to tissues. Nevertheless, triggering RBC transfusion by only Hb levels could be misleading since oxygen delivery to the tissues should be adapted according to the metabolic state and clinical condition.^(14)^ Conversely, microcirculatory blood flow distribution and tissue oxygen variables could better guide RBC transfusions, although early goal-directed therapy protocols, including RBC transfusion, have yielded contradictory clinical results in sepsis and septic shock.^(15-18)^

There is a paucity of data evaluating the effects of RBC transfusion on metabolic or tissue oxygen-derived parameters and microvascular blood flow variables, despite the large number of patients included in studies evaluating its clinical impact.^(19,20)^ Thus, we proposed this systematic review and meta-analysis to evaluate the impact of RBC transfusions on oxygen balance, tissue oxygenation and microcirculatory blood flow in patients with septic shock independently of the trigger or transfusion strategy used.

## METHODS

We conducted a systematic review and meta-analysis following the Cochrane recommendations^(21)^ and Preferred Reporting Items for Systematic Reviews and Meta-Analyses (PRISMA) guidelines^(22)^ with the PICO strategy: patients: adults admitted to intensive care units with diagnoses of sepsis or septic shock; intervention: RBC transfusion; comparison: usual care of these septic patients before RBC transfusions; outcome: pooled mean difference of mixed venous oxygen saturation (SvO_2_), oxygen extraction ratio (O_2_ER), cardiac index, near infrared spectroscopy (NIRS) parameters and sublingual microcirculation parameters by orthogonal polarization spectral (OPS) or side streamdark field (SDF) techniques before and after transfusion. The PRISMA checklist for systematic reviews and meta-analyses is provided in table 1S (Supplementary material).

### Study selection

Studies were included if they were performed in adult humans (aged 18 years or older) admitted to intensive care units for sepsis or septic shock without language restrictions. Studies were excluded if they were performed in healthy subjects, patients with hematologic diseases, Jehovah Witnesses or other patients who refused RBC transfusions. Studies performed for hemodilution analysis without transfusions, retransfusions or autotransfusions were also excluded.

Studies obtained for this systematic review reported data before and after RBC transfusions (quasi-experimental design) and therefore were included in the analysis. There were no randomized controlled trials retrieved through the search strategy. Case reports and medical communications were excluded.

### Outcomes

The main outcomes were the pooled mean differences in SvO_2_, O_2_ER, and cardiac index before and after transfusion. Detailed descriptions of each variable are presented in table 2S (Supplementary material). In addition, NIRS parameters (mean thenar tissue oxygenation - StO_2_%, tissue hemoglobin index - THI, thenar tissue oxygen saturation upslope of the reperfusion phase, thenar tissue oxygen saturation downslope), and sublingual microcirculation parameters (proportion of perfused small vessels - PPV, perfused small vessel density) were evaluated.

### Search strategy

Systematic search strategies followed established Cochrane and PRISMA recommendations. The literature search was performed using the National Center for Biotechnology Information (NCBI) and Elsevier databases from inception to July 2019. We used combined medical subject headings (MeSH) terms related to the intervention of interest (blood transfusions, RBC, erythrocyte transfusion) and outcomes of interest (SvO_2_, venous oxygen saturation, oxygen consumption, and microcirculation).

### Data collection process

Two investigators reviewed all of the titles and abstracts identified through systematic search strategies. Relevant titles or abstracts were retrieved as full texts. Articles selected for full-text review were independently reviewed by two investigators who determined their eligibility. Disagreements were resolved by a third reviewer.

The following information was extracted using a standardized data form: authors, year of publication, journal of publication, institution wherein the work was performed, study design, inclusion and exclusion criteria, sample size, clinical characteristics (Acute Physiology and Chronic Health Evaluation II - APACHE - II score, main pathology, transfusion trigger, number of RBC received), and clinical information regarding the outcomes of interest. All of the information was entered into an electronic database.

### Risk of bias

The internal validity of each study included in this systematic review was evaluated for bias according to the proposed evidence level of individual studies (ELIS) framework.^(23)^ Studies were classified from one (high quality or low risk of bias) to four (low quality or high risk of bias) according to study type, research design, and assessment of strength and limitations affecting the uncertainty of the results. One individual evaluated the quality and risk of bias of all of the studies using the ELIS framework.

### Data analysis

A meta-analysis was performed to assess the effect of RBC transfusions on oxygen delivery and cellular metabolism in critically ill septic patients. In addition, this meta-analysis attempted to assess the effect of volume expansion after RBC transfusions in the same population. Therefore, the percentage of SvO_2_, O_2_ER ratios, cardiac index and microcirculatory parameters were collected as continuous data. The analysis was restricted to available data collected within the systematic review.

Mean differences and 95% confidence intervals (95%CI) were pooled to estimate statistically significant differences before and after RBC transfusions in these critically ill septic patients. All meta-analyses were performed using a random-effects model (DerSimonian and Laird). Heterogeneity was evaluated using meta-regression estimates of between-study variance proportion of residual variation with the Knapp-Hartung modification (I-square test); heterogeneity was classified as low (I-square < 25%), medium (I-square = 25 - 75%), and high (I-square > 75%). Funnel plot asymmetry and Egger's test for small-study effects were performed using meta-regressions to examine whether the results of the meta-analyses may have been affected by publication bias or by the studies' Level of Evidence. P-values < 0.05 were considered statistically significant. All analyses were performed using Stata (version 15) software.

## RESULTS

### Characteristics of the included studies

**Figure 1:**
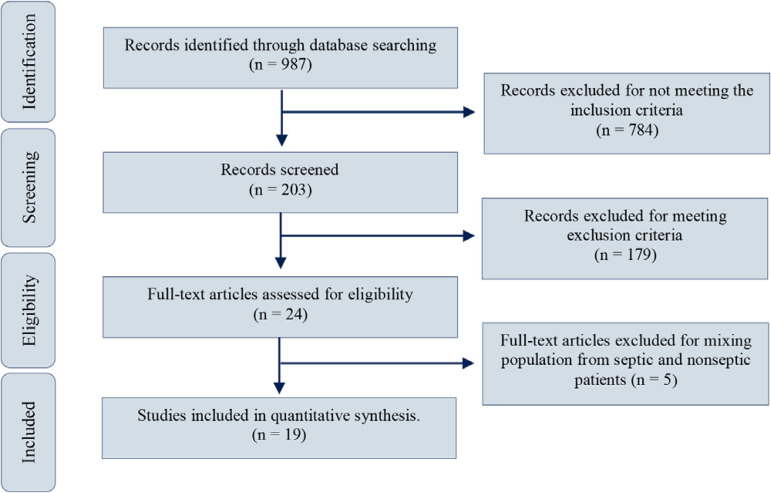
Study selection.

Source: adapted from Moher D, Liberati A, Tetzlaff J, Altman DG; PRISMA Group. Preferred reporting items for systematic reviews and meta-analyses: the PRISMA statement. PLoS Med. 2009;6(7):e1000097.^(22)^

Nine hundred eighty-seven (987) manuscripts published between July 1968 and July 2019 were obtained after entering keywords in the MEDLINE^®^, Embase and Scopus search boxes. Twenty-four full-text articles were assessed for eligibility; of these, 5 articles were excluded because of a mixed population of septic and nonseptic patients. Nineteen studies comprising data from 428 patients before and after RBC transfusions were finally included in the qualitative and quantitative analyses (Figure 1). The general characteristics of these studies are presented in tables 3SA, 3SB, 3SC, 4SA and 4SB (Supplementary material).

Data from the subgroup analysis performed by Gilbert et al.,^(24)^ Silverman et al.,^(25)^ Sadaka et al.,^(26)^ and Mazza et al.^(14)^ were abstracted and analyzed separately because we considered that each subgroup represented a different population.

### Risk of bias

The Level of Evidence analyzed through the ELIS system revealed that 5 studies met the criteria for level II evidence,^(11,14,26-28)^ 8 studies were classified as level III,^(12,24,25,29-33)^ and the remaining were level IV (Table 5S - Supplementary material).

### Outcomes assessment

#### Mixed venous oxygen saturation

**Figure 2:**
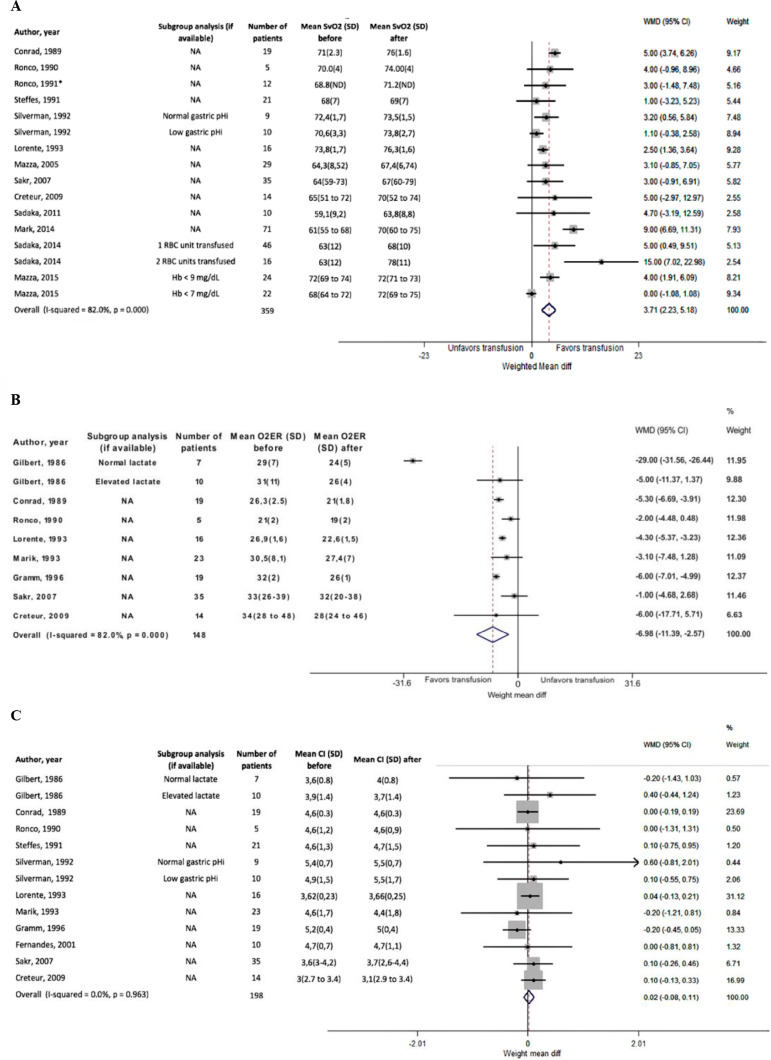
Unstandardized mean differences.

There were 359 patients from 14 studies with information on SvO_2_ before and after RBC transfusions. Red blood cell transfusions were associated with an increase in the pooled mean SvO_2_ of 3.7% (95%CI, 2.23 - 5.18, p < 0.001) (Table 1, Figure 2A). Nonetheless, heterogeneity was high (I-square = 82%). Funnel plot asymmetry and Egger's test for small-study effects demonstrated a lack of publication bias (p = 0.155) (Figure 1SA - Supplementary material). No significant differences were observed for different Levels of Evidence in the meta-regression analysis (p = 0.334) (Figure 2SA - Supplementary material).

**Table 1:** Unstandardized mean differences in mixed venous oxygen saturation, oxygen extraction ratio, cardiac index and microcirculatory parameters before and after red blood cell transfusions

Outcome	Number of studies	Number of patients	Weight mean difference (95%CI)	p value	I-squared (%)
Unstandardized mean differences in SvO_2_	14	359	3.71 (2.23 - 5.18)	< 0.001	82
Unstandardized mean differences in O2ER	8	148	-6.98 (-11.39 - -2,57)	< 0.001	82
Unstandardized mean differences in CI	7	198	0.02 (-0.08 - 0.11)	0.96	0
Unstandardized mean differences in thenar tissue oxygen	5	69	1.13 (-1.14 - 3.40)	0.894	0
Unstandardized mean differences tissue Hb Index	5	69	1.66 (0.05 - 3.26)	0.018	66.6
Unstandardized mean differences in thenar tissue oxygen saturation upslope of the reperfusion phase	5	69	0.17 (-0.22 - 0.55)	0.822	0
Unstandardized mean differences in thenar tissue oxygen saturation downslope	4	48	0.56 (-0.41 - 1.54)	0.933	0
Unstandardized mean differences in proportion of perfused small vessels (%)	5	76	2.85 (1.22 - 4.47)	0.553	0
Unstandardized mean differences in pPerfused small vessel density (mm/mm^2^)	5	76	1.19 (-0.58 - 2.96)	0.044	59.2

95%CI - 95% of confidence interval; SvO_2_ - mixed venous oxygen saturation; O2ER - oxygen extraction ratio; CI - cardiac index; Hb - hemoglobin.

(A) Mixed venous oxygen saturation, (B) oxygen extraction ratio and (C) cardiac index. All studies included.

SvO_2_ - mixed venous oxygen saturation; SD - standard deviation; WMD - weight mean difference; 95%CI - 95% of confidence interval; RBC - red blood cells; O_2_ER - oxygen extraction ratio; CI - cardiac index.

#### Oxygen extraction ratio

There were 148 patients from 8 studies with information on O_2_ER before and after RBC transfusions. In the meta-analysis, pooled mean differences demonstrated a statistically significant decrease in the O_2_ER of -6.98 (95%CI, -11.39 - -2.57; p < 0.001) (Table 1, Figure 2B). Again, the heterogeneity was high (I-squared = 82%). Funnel plot asymmetry and Egger's test for small-study effects demonstrated a lack of publication bias (p = 0.674) (Figure 1SB - Supplementary material). No significant differences were observed for different levels of evidence in the meta-regression analysis (p = 0.171) (Figure 2SB - Supplementary material).

#### Cardiac index

There were 198 patients from seven studies with information on the cardiac index before and after RBC transfusions. In summary, all of the studies reported similar cardiac indexes before and after RBC transfusions (Table 6S - Supplementary material). The pooled mean difference in the cardiac index before and after RBC transfusion was 0.02 (95%CI -0.08 - 0.11, p = 0.96) (Table 1, Figure 2C), while the heterogeneity was low (0.0%). Funnel plot asymmetry and Egger's test for small-study effects demonstrated a lack of publication bias (p = 0.518) (Figure 1SC - Supplementary material). No significant differences were observed for different Levels of Evidence in the meta-regression analysis (p = 0.436) (Figure 2SC - Supplementary material).

**Figure 3:**
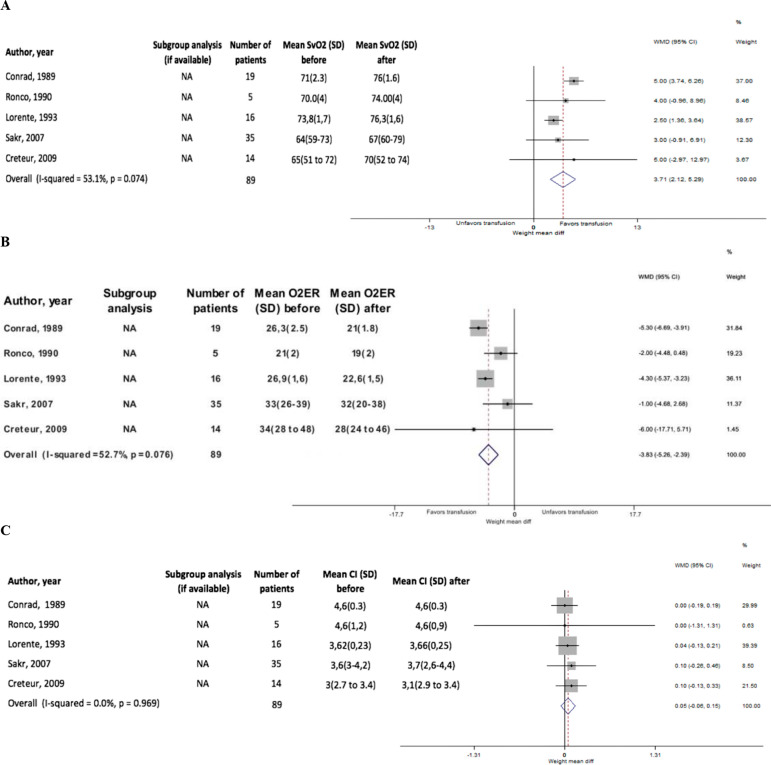
Unstandardized mean differences.

**Figure 4:**
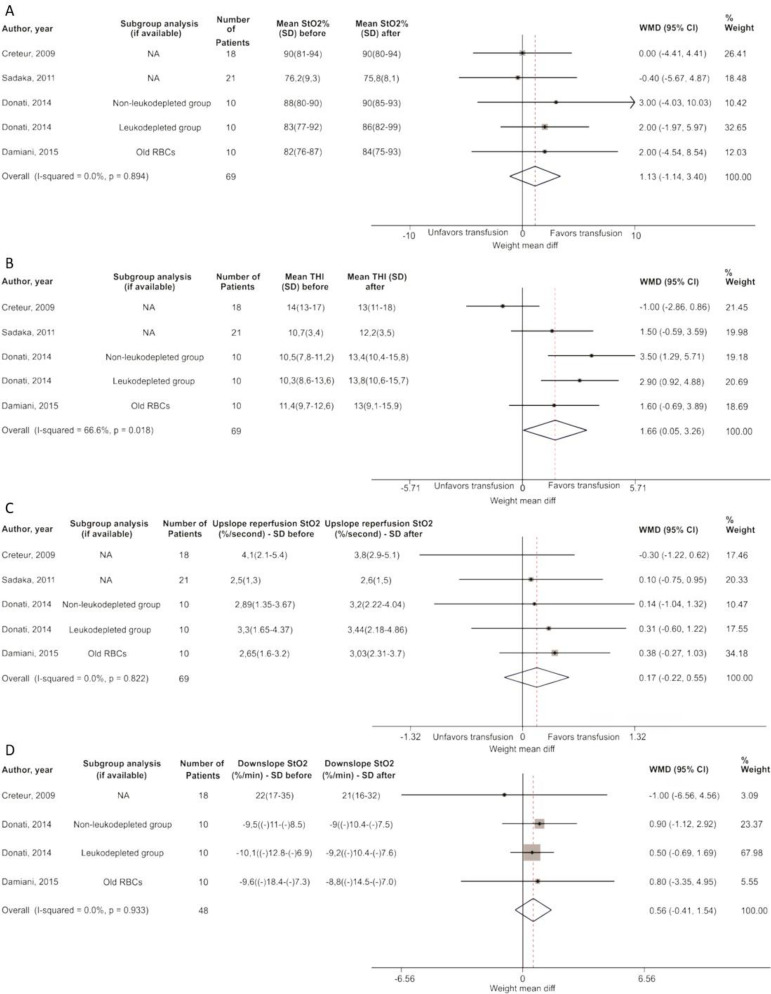
Unstandardized mean differences.

There were 75 patients in which SvO_2_, O_2_ER, and cardiac index were simultaneously measured. The pooled mean difference of SvO_2_ in this group demonstrated a statistically significant SvO_2_ increase of 3.71% (95%CI 2.12 - 5.29; p = 0.074; I-square=53.1%), a statistically significant decrease in O_2_ER of -3.83 (95%CI -5.26 - -2.39; p = 0.076; I-square = 52.7%), and a pooled mean difference in CI of 0.05 (95%CI -0.06 - 0.15; p = 0.096; I-square = 0.0%) (Figure 3). Funnel plot asymmetry and Egger's test for small-study effects demonstrated a lack of publication bias (Figure 3S - Supplementary material). No significant differences were observed for different Levels of Evidence in the meta-regression analysis (Figure 4S - Supplementary material).

(A) Mixed venous oxygen saturation, (B) oxygen extraction ratio and (C) cardiac index of studies reporting simultaneously mixed venous oxygen saturation, oxygen extraction ratio and cardiac index. SvO_2_ - mixed venous oxygen saturation; SD - standard deviation; WMD - weight mean difference; 95%CI - 95% of confidence interval; O_2_ER - oxygen extraction ratio; CI - cardiac index.

### Near infrared spectroscopy

A group of 69 patients were measured for StO_2_, THI, upslope reperfusion StO2 and downslope StO2. Thenar tissue oxygen saturation revealed a pooled mean difference of 1.13% (95%CI -1.14 - 3.40; p = 0.894; I-square = 0.0%) and an increase in THI of 1.66 (95%CI 0.05 - -3.26; p = 0.018; I-square = 66.6%) after RBC transfusion. Meanwhile, the pooled mean difference in the up- and downslopes of StO_2_ during reperfusion did not show significant variations after RBC transfusion (Table 1, Figure 4).

(A) Thenar tissue oxygen, (B) tissue hemoglobin index, (C) thenar tissue oxygen saturation upslope of the reperfusion phase and (D) thenar tissue oxygen saturation downslope (n = 75). StO_2_ - thenar tissue oxygen saturation; SD - standard deviation; WMD - weight mean difference; 95%CI - 95% of confidence interval; RBC - red blood cells; THI - tissue hemoglobin index.

Creteur et al.^(13)^ calculated the NIRS-derived tissue oxygen consumption (NIRS VO_2_) before and after RBC transfusions (∆NIRS VO_2_). In this study, RBC transfusion did not globally affect NIRS-derived variables. However, ∆NIRS VO_2_ had a weak but significant relationship with the ΔStO_2_ upslope during the reperfusion phase (r2 = 0.14; p = 0.038). In addition, ΔStO_2_ upslope during the reperfusion phase had a negative correlation with the baseline StO_2_ upslope (r2 = 0.42; p < 0.0001) (Table 6S - Supplementary material).

Similarly, another study^(11)^ demonstrated that RBC transfusion did not globally affect NIRS-derived variables, although ∆NIRS VO_2_ was negatively correlated with the baseline (r = -0.679, p = 0.001). In addition, there was a positive correlation between ∆NIRS VO_2_ and the % change in the StO_2_ recovery upslope (r = 0.442, p = 0.045) (Table 6S - Supplementary material).

### Sublingual microcirculation

**Figure 5:**
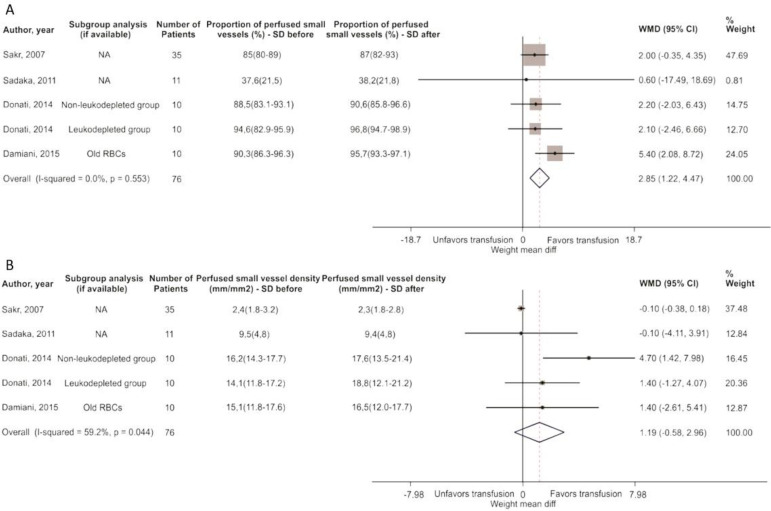
Unstandardized mean differences.

A group of 76 patients were measured for the PPV and the density of small vessels perfused or their functional capillary density (FCD), either by SDF or OPS techniques. The PPV revealed a significant increase of 2.85% (95%CI 1.22 - 4.47; p = 0.553; I-square = 0.0%), while the FCD showed a significant increase of 1.19 (95%CI -0.58 - 2.96; p = 0.044; I-square = 59.2%) (Table 1, Figure 5).

(A) The proportion of perfused small vessels (%) and (B) perfused small vessel density (mm/mm^2^).

SD - standard deviation; WMD - weight mean difference; 95%CI - 95% of confidence interval; RBC - red blood cells.

Sakr et al.^(12)^ described the evolution of microcirculatory variables before and after RBC transfusion in patients with normal *versus* altered capillary perfusion at baseline. In this study, patients with abnormal capillary perfusion at baseline had a greater and significant increase in PPV (4.5 [3.6 - 4.9] to 4.8 [3.6 - 4.9], p < 0.05, *versus* 5.2 [4.3 - 5.7] to 5.1 [4.4 - 5.7], p > 0.05) and FCD (2.7 [1.9 - 3.0] to 3.2 [2.7 - 3.9], p < 0.01, *versus* 3.5 [3.2 - 4.5] to 3.3 [3.0 - 3.8], p > 0.05) than patients with normal microcirculation at baseline (Table 6S - Supplementary material).

## DISCUSSION

This systematic review and meta-analysis suggests there are positive effects of RBC transfusions on metabolic oxygen variables (SvO_2_ and O_2_ER) in patients with sepsis and septic shock, with apparent independence from macrocirculatory changes. Additionally, RBC transfusions positively impact microcirculation and tissue oxygen consumption, especially when the microcirculation and/or tissue VO_2_ are altered at baseline. Although RBC transfusions globally increased THI, it was not possible to demonstrate a significant impact on StO_2_ or microvascular vasoreactivity variables. Most papers included in this review and meta-analysis used Hb levels as a transfusion trigger, but remarkably, the studies using low SvO_2_ or altered tissue perfusion signs to trigger RBC transfusion were those demonstrating the maximal benefit on oxygen metabolic parameters and microvascular blood flow.

Traditionally, Hb levels have been proposed as triggers for RBC transfusion.^(5-10)^ In fact, conservative transfusion strategies are based on the assumption that maintaining a minimal Hb level should ensure adequate oxygen transport to tissues and thus adequate cell metabolism. Data coming from animal experimental models suggest that extreme hemodilution is well tolerated until Hb concentrations fall to 30 or 50g/L, when depressed left ventricular function and ischemic electrocardiographic changes occur, respectively.^(34)^ Similar results were obtained in resting healthy volunteers subjected to acute isovolemic hemodilution up to Hb concentrations of 50g/L,^(35)^ suggesting a good tolerance to hemodilution anemia in a wide range of Hb levels. However, such tolerance is not as good in the presence of coronary stenosis, with myocardial ischemia signs appearing earlier.

Clinical evidence revealed that restrictive transfusion strategies could be as safe as liberal practices, except in patients with myocardial ischemia.^(5)^ Consequently, restricted transfusion became a standard of care in the intensive care unit. However, only the Transfusion Requirements in Septic Shock trial^(9)^ compared restrictive *versus* liberal strategies of RBC transfusion in septic patients, demonstrating no significant differences in mortality at day 90. Nevertheless, previously transfused patients were excluded, which reduces its external validity.^(9)^ Alternatively, observational studies about the impact of RBC transfusion in septic patients reveal contradictory results on clinical outcomes.^(36-39)^ Nevertheless, no physiological oxygen metabolic parameters were the studied outcomes in any of these studies.

Triggering RBC transfusion based on Hb levels could be misleading since oxygen delivery should be adapted according to the metabolic state and clinical condition.^(14)^ In fact, transfusions performed in the TRISS trial^(9)^ and other cohort studies^(36-38)^ were mostly triggered by Hb levels and were not restricted to the early stages of resuscitation, which probably hampered any potential benefit of RBC transfusion. Our results suggest that RBC transfusions could be favorable when abnormal oxygen metabolic parameters are previously altered. Unfortunately, data from our meta-analysis are not able to clarify whether patients showing improvements in oxygen variables would eventually have some clinical outcome benefit.

Early studies suggested that blood transfusions increased oxygen delivery to the tissues in septic patients.^(24,40)^ However, subsequent papers failed to demonstrate significant increases in SvO_2_ following RBC transfusion.^(30,41-44)^ Nevertheless, blood transfusions are recommended as a therapeutic intervention to increase SvO_2_ when other strategies fail to do so.^(45)^ Resuscitation bundles incorporated into early goal-directed protocols (EGDT) included RBC transfusions as a therapeutic intervention aiming to achieve SvO_2_ > 70%. While an initial study of EGDT demonstrated a favorable impact on mortality rates,^(15)^ later studies were not able to demonstrate any significant benefit.^(16-18)^ However, rates of RBC transfusion in these studies were relatively low: 8.8% *versus* 3.8% for EDGT *versus* control in the ProMISe trial;^(16)^ 13.6% *versus* 7.0% for EDGT *versus* control in the ARISE trial,^(17)^ and 14.4% *versus* 7.5% for EDGT *versus* control in the ProCESS trial.^(18)^ Furthermore, the SvO_2_ reported at baseline in the EGDT groups was 70%, 73% and 71% in the ProMISe, ARISE and ProCESS studies, respectively.^(46)^ Even when very early phases of septic shock are considered, these last few clinical trials were not able to identify patients potentially benefiting from a higher transfusion threshold.

Although hematocrit levels could positively affect oxygen transport (DO_2_) at a macrocirculatory level, i.e., improving SvO_2_ and O_2_ER variables, it remains unclear how an increased hematocrit could influence microcirculatory DO_2_.^(47-49)^ Some authors have suggested that microcirculatory stagnation and impaired DO_2_ to the tissues might be closely related to hematocrit variations, theorizing that normovolemic hemodilution can improve microcirculation and DO_2_,^(47,50)^ while others have suggested the limited effects of the hematocrit on microcirculation.^(51)^ Nevertheless, recent observations in experimental septic shock demonstrated a close relationship between microvascular blood flow distribution at the jejunal villi and the dynamic variations of regional mesenteric O_2_ER, thus suggesting a close link between microvascular blood flow distribution and tissue oxygenation during the early stages of septic shock.^(52)^ Additionally, other observations suggest that RBC transfusions positively impact the lactate/pyruvate ratio in septic patients.^(53)^

Red blood cell transfusions globally improve microcirculation by increasing convective blood flow. Interestingly, this effect is more evident when the baseline microcirculatory perfusion is more altered.^(12)^ Accordingly, oxygen tissue consumption was improved after RBC transfusion only among patients with low NIRS-VO_2_ or altered microvascular reactivity at baseline.^(11,13)^ Nevertheless, data on tissue oxygen variables after RBC transfusion are scarce, and our conclusions are based on a limited number of patients.

Finally, some studies investigated the effect of RBC transfusion according to storage and leukocyte reduction. Although, in general, no differences in microvascular perfusion were observed between leukodepleted and non-leukodepleted RBC transfusions, the microvascular flow index and blood flow velocity showed some superiority with leukodepleted RBCs, suggesting a possible beneficial effect of this modality on convective flow in the microcirculation. A secondary analysis of this study^(33)^ described how transfusion of fresh *versus* old RBCs had a positive and significant impact on NIRS-derived microcirculatory parameters in septic patients. However, neither fresh nor old RBCs improved sublingual microcirculation following transfusion.^(33)^

Our systematic review and meta-analysis has important limitations. First, we did not have access to the original raw data, so this meta-analysis used the data reported in each of the papers included. Consequently, regrouping patients into more biologically plausible subgroups was not possible. Second, our study has the same "baseline risk" as the pooled research analysis, in which patients with different risks may fare differently when exposed to the same intervention. In our case, it is impossible to determine whether RBC transfusion could have better benefits since the baseline characteristics of sepsis and septic shock differed widely among studies. Nevertheless, most studies agreed that the more altered the microcirculatory blood flow and the lower the NIRS-VO_2_ were, the more beneficial RBC transfusion was. Third, our conclusions are entirely based on studies with considerable heterogeneity in which RBCs were presupposed to be beneficial. Fourth, even though our data suggest a potential biological benefit mediated by RBC transfusions, they do not support the idea that improving oxygen metabolic parameters or microcirculatory convective flow can modify clinical outcomes in septic shock.

## CONCLUSION

Red blood cell transfusions seem to improve systemic oxygen metabolism in patients with sepsis and septic shock, with apparent independence from cardiac index variations. Red blood cell transfusions apparently improve some tissue oxygenation and microcirculation parameters, particularly in patients with baseline abnormalities. More studies are necessary to evaluate their clinical impact and to individualize transfusion decisions.
