# Supplementary material for: Impact of red blood cell transfusion on oxygen transport and metabolism in patients with sepsis and septic shock: a systematic review and meta-analysis
Source: Rev Bras Ter Intensiva. 2021 Jan-Mar;33(1):154–66. doi: 10.5935/0103-507X.20210017 (PMC8075342; doi:10.5935/0103-507X.20210017)
Supplement: Supplementary file 1 [file rbti-33-01-0154-suppl1.pdf]

Impact of red blood cell transfusion on oxygen transport and metabolism in patients with sepsis and septic shock: a systematic review and meta-analysis

Impacto da transfusão de eritrócitos no transporte e no metabolismo de oxigênio em pacientes com sepse e choque séptico: uma revisão sistemática e metanálise

María Camila Arango-Granados[1,2], Mauricio Umaña[1,2], Álvaro Ignacio Sánchez[1,2], Alberto Federico García[1,2], Marcela Granados[1,2], Gustavo Adolfo Ospina-Tascón[1,2]

Table 1S - Preferred Reporting Items for Systematic Reviews and Meta-Analyses (PRISMA) checklist for systematic reviews and meta-analysis

Section/topic	#	Checklist item	Reported on page #
**TITLE**			
Title	1	Identify the report as a systematic review, meta-analysis, or both	1
**ABSTRACT**			
Structured summary	2	Provide a structured summary including, as applicable: background; objectives; data sources; study eligibility criteria, participants, and interventions; study appraisal and synthesis methods; results; limitations; conclusions and implications of key findings; systematic review registration number	2 - 3
**INTRODUCTION**			
Rationale	3	Describe the rationale for the review in the context of what is already known	4
Objectives	4	Provide an explicit statement of questions being addressed with reference to participants, interventions, comparisons, outcomes, and study design (PICOS)	5
**METHODS**			
Protocol and registration	5	Indicate if a review protocol exists, if and where it can be accessed (e.g., Web address), and, if available, provide registration information including registration number	N.A.
Eligibility criteria	6	Specify study characteristics (e.g., PICOS, length of follow-up) and report characteristics (e.g., years considered, language, publication status) used as criteria for eligibility, giving rationale	5 - 6
Information sources	7	Describe all information sources (e.g., databases with dates of coverage, contact with study authors to identify additional studies) in the search and date last searched	6
Search	8	Present full electronic search strategy for at least one database, including any limits used, such that it could be repeated	6
Study selection	9	State the process for selecting studies (i.e., screening, eligibility, included in systematic review, and, if applicable, included in the meta-analysis)	6
Data collection process	10	Describe method of data extraction from reports (e.g., piloted forms, independently, in duplicate) and any processes for obtaining and confirming data from investigators	6 - 7
Data items	11	List and define all variables for which data were sought (e.g., PICOS, funding sources) and any assumptions and simplifications made	6 - 7
Risk of bias in individual studies	12	Describe methods used for assessing risk of bias of individual studies (including specification of whether this was done at the study or outcome level), and how this information is to be used in any data synthesis	7
Summary measures	13	State the principal summary measures (e.g., risk ratio, difference in means)	7 - 8
Synthesis of results	14	Describe the methods of handling data and combining results of studies, if done, including measures of consistency (e.g., I2) for each meta-analysis	7 - 8
Risk of bias across studies	15	Specify any assessment of risk of bias that may affect the cumulative evidence (e.g., publication bias, selective reporting within studies)	7 - 8
Additional analyses	16	Describe methods of additional analyses (e.g., sensitivity or subgroup analyses, meta-regression), if done, indicating which were pre-specified	7 - 8
**RESULTS**			
Study selection	17	Give numbers of studies screened, assessed for eligibility, and included in the review, with reasons for exclusions at each stage, ideally with a flow diagram	8
Study characteristics	18	For each study, present characteristics for which data were extracted (e.g., study size, PICOS, follow-up period) and provide the citations	8 - 10
Risk of bias within studies	19	Present data on risk of bias of each study and, if available, any outcome level assessment (see item 12)	12
Results of individual studies	20	For all outcomes considered (benefits or harms), present, for each study: (a) simple summary data for each intervention group (b) effect estimates and confidence intervals, ideally with a forest plot	11 - 15
Synthesis of results	21	Present results of each meta-analysis done, including confidence intervals and measures of consistency	11 - 15
Risk of bias across studies	22	Present results of any assessment of risk of bias across studies (see Item 15)	11 - 15
Additional analysis	23	Give results of additional analyses, if done (e.g., sensitivity or subgroup analyses, meta-regression – see Item 16)	11 - 15

Continue...

...continuation

**DISCUSSION**			
Summary of evidence	24	Summarize the main findings including the strength of evidence for each main outcome; consider their relevance to key groups (e.g., healthcare providers, users, and policy makers)	16
Limitations	25	Discuss limitations at study and outcome level (e.g., risk of bias), and at review-level (e.g., incomplete retrieval of identified research, reporting bias)	19
Conclusions	26	Provide a general interpretation of the results in the context of other evidence, and implications for future research	19
**FUNDING**			
Funding	27	Describe sources of funding for the systematic review and other support (e.g., supply of data); role of funders for the systematic review	

PICOS - Patient, Intervention, Comparison, Outcome.

Table 2S - Abbreviations and basic principles

Formula	Expressed as
$CO = VO_2 / Ca\text{-}vO_2$	L/minute
$CI = CO$ / Body surface area	L/minute/Mts^2
$CaO_2 = Hb \times SaO_2 \times 1.39$	
$CvO_2 = Hb \times SvO_2 \times 1.39$	
$= CaO_2 - (VO_2 / CO)$	mL/dL
$VO_2 = CI \times (Ca\text{-}vO_2) \times 10$	
$= CI \times Hb\ (SaO_2 – SvO_2)\ 13.9$	mL/minute
$DO_2 = CO \times CaO_2 \times 10$	mL/minute
$O_2ER = (SaO_2 - SvO_2) / SaO_2$	
$= VO_2 / DO_2$	Ratio
$SvO_2 = SaO_2 - (VO_2 / CI \times Hb \times 13.9)$	%
NIR VO2 = [(StO2 desaturation slope-1) × (THIstart cuff + THI1 min)] / 2	

CO - cardiac output; VO_2 - oxygen consumption; $Ca\text{-}vO_2$ - arteriovenous oxygen difference; CI - cardiac index; CaO_2 - arterial oxygen content; Hb - hemoglobin; SaO2 - arterial oxygen saturation; CvO_2- venous oxygen content; CI - cardiac index; DO_2 - oxygen delivery; O_2ER - oxygen extraction ratio; SvO_2 - mixed venous oxygen saturation; NIR VO_2 - near infrared spectroscopy derived tissular oxygen consumption; StO_2 - tissular oxygen saturation; THI - tissue hemoglobin index.

Table 3SA - General characteristics of the studies reporting mixed venous oxygen saturation, oxygen extraction ratio and cardiac index

Author	Journal	Institution	Study Design	Time analyzed
Sadaka et al.[11]	Annals of Intensive Care	ND	Prospective, observational study	ND
Sakr et al.[12]	Critical Care of Medicine	Erasme Hospital	Prospective, observational study	ND
Mazzaet al.[14]	Revista Brasileira de Terapia Intensiva	Three Brazilian general ICU	Multi-center, prospective, randomized study, not blinded	March 1st and August 31st 2008
Gilbert et al.[24]	American Review of Respiratory Disease	Detroit Receiving Hospital, Harper Hospital, Critical Care Center of Mount Carmel Mercy Hospital	Prospective clinical trial	Sept 1981 - Jan 1985
Silverman et al.[25]	Chest	ND	Retrospective review	ND
Sadaka et al.[26]	Journal of Clinical Medicine Research	ND	Retrospective review	June 2011 and March 2013
Mark et al.[27]	Critical Care	Kaiser Permanente Northern California	Retrospective review of a prospective quality improvement database	March 2010 - September 2012
Fernandes et al.[28]	Critical Care	ND	ND	ND
Steffes et al.[30]	Critical Care of Medicine	Harper Hospital, Detroit Receiving Hospital	Prospective study	ND
Ronco et al.[31]*	American Review of Respiratory Disease	St. Paul's Hospital	Prospective study	October 1988 - December 1989
Marik et al.[32]	JAMA	Tertiary care teaching hospital	Prospective, controlled, interventional study	ND
Ronco et al.[40]	Chest	St. Paul´s Hospital	Prospective study	June 1988 - June 1989
Conrad et al.[41]	Circulatory Shock	Medical and Pediatric ICUs of Louisiana State University Medical Center	Prospective	ND
Lorente et al.[42]	Critical Care of Medicine	Tertiary care hospital	Prospective, randomized, interventional crossover study.	ND
Gramm et al.[43]	Shock	Surgical Intensive Care Unit	Prospective study	ND
Mazzaet al.[44]	Clinics	General ICU	Prospective	February 2001 to October 2002

ND - no data; RBC - red blood cell. *Subgroup of septic patients.

Table 3SB - General characteristics of the studies reporting mixed venous oxygen saturation, oxygen extraction ratio and cardiac index

Author	Primary endpoints	Secondary endpoints	Inclusion criteria	Exclusion criteria
Sadaka et al.[11]	Evaluate the effect of RBC transfusion in severe septic patients on sublingual microvascular perfusion and flow using SDF and on muscle tissue oxygenation, oxygen consumption, and microvascular reactivity using NIRS	Correlate the variables obtained from NIRS with those obtained from SDF. In-hospital mortality	Severe sepsis, clinically euvolemic (by CVP and/or echocardiogram) and in the first 12 hours of sepsis, with hemoglobin < 7.0, or for a hemoglobin between 7.0 and 9.0 with lactic acidosis, or central venous oxygen saturation < 70%	RBC transfusion in the preceding 72 hours, peripheral vascular disease, liver cirrhosis, age < 18 years, active bleeding, shock secondary to any other cause (cardiogenic, hemorrhagic, obstructive), and pregnancy
Sakr et al.[12]	Hemodynamic and microvideoscopic assessments (OPS)	ND	Patients requiring transfusion, with severe sepsis, considered euvolemic, mechanically ventilated	Liver cirrhosis, RBC transfusion in the preceding 72 hours, shock owing to any other cause (cardiogenic, hemorrhagic, obstructive), oral injuries, rapid deterioration of hemodynamic status with the need to increase vasopressor dose in the 2 hours preceding transfusion, previous inclusion in the study
Mazza et al.[14]	Hb levels, $ScvO_2$ and lactate	ND	Age over 18 years old, a shock diagnosis that was made less than 48 hours prior to participation in the study, Hb levels 9.0 - 7.0g/dL and a central venous catheter in the superior vena cava	Pregnancy, known coronary disease, active bleeding and previous participation in the study
Gilbert et al.[24]	Oxygen delivery and oxygen consumption	ND	Septic, normal or elevated lactate	If during the trial they presented: temperature change > 1°C, agitation or restlessness, seizures, clinical signs suggesting changes in respiratory work, sudden deterioration of clinical status (cardiopulmonary arrest or gastrointestinal bleeding)
Silverman et al.[25]	Gastric pHi	MAP, CO, SaO_2, SvO_2, Hb, lactate, DO_2, VO_2	Sepsis syndrome, normal or low pHi	ND
Sadaka et al.[26]	SvO_2 and mortality	Early goal achievement	Once patient is diagnosed with SS, a 20mL/kg bolus of crystalloid was given to achieve a central venous pressure of 8 - 12mmHg. If the mean arterial pressure was less than 65mmHg, vasopressors were given to maintain a mean arterial pressure of at least 65mmHg. Once these goals are achieved, if the central venous oxygen saturation was less than 70%, RBCs were transfused to achieve a hematocrit of at least 30%	ND
Mark et al.[27]	In-hospital mortality	Total intravenous fluids, hemoglobin nadir, vasopressor administration, initiation of mechanical ventilation, percent lactate clearance per hour, shock index, 6-hour mSOFA score, Elixhauser score, and final $ScvO_2 \geq 70\%$	Two or more $ScvO_2$ measurements less than 70% during the first 6 hours of EGDT eligibility along with adequate arterial oxygenation ($SpO_2 \geq 93\%$) and concomitant (within 15 minutes) CVP and MAP values that were at or above target goals	Patients under the age of 18 or with pregnancy were excluded from the prospective EGDT database
Fernandes et al.[28]	Hemodynamic data, gastric tonometry and calorimetry	ND	Septic patients with Hb <10mg/dL, mechanically ventilated, with an FIO_2 less than 60% and with pulmonary artery catheter monitoring	Age < 18 or > 80 years, pregnant women, patients on dialysis, patients who had recently undergone a gastrointestinal surgical procedure, established septic shock
Steffes et al.[30]	Lactate, DO_2, VO_2	Hemodynamic parameters	Postoperative or post-trauma, admitted to the ICU, with sepsis diagnosis and hypotension (BP < 80mmHg), SVR < 800, unexplained metabolic acidosis or suddenly increasing fluid requirement, hemodynamically stable	Changes in pre and post-transfusion pulmonary artery occlusion pressure > 3 mmHg, SaO_2 or inotrope dose
Ronco et al.[31]*	VO_2, DO_2	CO, Hb, PaO_2, PvO_2, SaO_2, SvO_2	Mechanically ventilated, paralyzed, and all of the following criteria for diagnosis of ARDS: arterial oxygen tension less than or equal to 75mmHg while breathing at least 50% oxygen, bilateral diffuse pulmonary infiltrates on chest roentgenogram, and a pulmonary artery occlusion pressure less than 18mmHg	Myocardial infarction within the previous 6 months

Continue...

...continuation

Marik et al.[32]	Gastric pHi, hemodynamic parameters	ND	Septic patients, who had a Hb concentration of less than 100g/L and were receiving mechanical ventilation	Dialysis requirement, < 18 years of age, recent upper gastrointestinal surgery, if the study physicians felt they were unlikely to survive longer than 24 hours following initiation of the study, established septic shock
Ronco et al.[40]	$DO_2$1, VO_2I, O_2ER	CI, Hb, PaO_2, PvO_2, SaO_2, SvO_2	Acute respiratory failure secondary to AIDS related Pneumocystis carinni pneumonia, admitted to the ICU, with a bronchoscopic diagnosis of PCP (first episode), a hemoglobin concentration less than 126g/dL, and a pulmonary artery catheter inserted within 24 hours of study	ND
Conrad et al.[41]	CO, PAWP, RAP, CI, CaO_2, CvO_2, DO_2, VO_2, O_2ER	ND	Septic shock, hemodynamically stable	ND
Lorente et al.[42]	MAP, MPAP, RAP, PAWP, CI, SVR, PVR, Hb, SaO_2, SvO_2, DO_2, VO_2, O_2ER	ND	Severe sepsis and Hb < 10mg/dL	Changes in core temperature > 0.5°C or with a bacteremic episode during interventions
Gramm et al.[43]	CO, hemodynamic monitoring	ND	Septic, volume-resuscitated acutely ill patients monitored invasively during their stay in the surgical ICU	ND
Mazza et al.[44]	SvO_2 and serum lactate	ND	>15 years, SIRS/sepsis, Hb < 9, invasive hemodynamic monitoring with OPAP > 12mmHg	Pregnancy, brain death, expected death in less than 24 hours

RBC - red blood cells; SDF - sidestream dark field; NIRS - near infrared spectroscopy; CVP - central venous pressure; OPS - orthogonal polarization spectral; ND - no data; Hb - hemoglobin; $ScvO_2$ - central venous oxygen saturation; pHi - intramucosal pH ; MAP - mean arterial pressure; CO - cardiac output; SaO_2 - arterial oxygen saturation; SvO_2 - mixed venous oxygen saturation; DO_2 - oxygen delivery; VO_2 - oxygen consumption; EGDT - ; FIO_2 - oxygen inspired fraction; ICU - intensive care unit; BP - blood pressure; SVR - systemic vascular resistance; PaO_2 - arterial oxygen partial pressure; PvO_2 - venous oxygen partial pressure; CI - cardiac index; PCP - *Pneumocystis carinni pneumonia*; PAWP - pulmonary artery wedge pressure; RAP - right atrial pressure; CaO_2 - arterial oxygen content; CvO_2 - venous oxygen content; O_2ER - oxygen extraction ratio; MPAP - mean pulmonary arterial pressure; PVR - pulmonary vascular resistance . *Subgroup of septic patients.

Table 3SC - General characteristics of the studies reporting mixed venous oxygen saturation, oxygen extraction ratio and cardiac index

Author	Number of patients	Main pathology	Mean APACHE II at admission	Transfusion trigger	Number of RBC received (mean)
Sadaka et al.[11]	10	Severe sepsis	24	Hemoglobin < 7.0, or for a hemoglobin between 7.0 and 9.0 with lactic acidosis, or central venous oxygen saturation < 70%	ND
Sakr et al.[12]	35	Severe sepsis and septic shock	25	Hb < 7 g/dL or between 7 and 9g/dL in the presence of signs of altered tissue perfusion	1 - 2
Mazza et al.[14]	24	Sepsis and: HTA, CAD, CHF, DM, CKD, CPD, CHC, Immunosuppression, neoplasm, CVD	13.9	Hb < 9	ND
Gilbert et al.[24]	7	Sepsis	ND	Circulatory failure and Hb < 10mg/dL	ND
Silverman et al.[25]	9	The source of infection was lung in 10 patients, kidney in 5, and an empyema, catheter related infection, endocarditis, epidural abscess, peritonitis, and osteomyelitis in the other 6 patients	ND	ND	ND
Sadaka et al.[26]	46	Severe sepsis	ND	SvO2 < 70%	1
Mark et al.[27]	71	Sepsis	ND	SvO2 < 70%	ND
Fernandes et al.[28]	10	Sepsis	25.5	Hb < 10	1
Steffes et al.[30]	21	Sepsis and: trauma, abdominal abscess, ulcer hemorrhage, gastric carcinoma, intestinal ischemia, burns, necrotizing fasciitis, Fournier´s gangrene, esophageal perforation, colon perforation, ulcer perforation, laryngeal cancer	21	Hypotension (BP< 80mmHg), SVR < 800, unexplained metabolic acidosis or suddenly increasing fluid requirement	ND

Continue...

...continuation

Ronco et al.[31]*	12	Pulmonary sepsis, chorioamnionitis, alcoholic ketoacidosis, septic shock	21	ND	600mL of packed RBC (70 percent hematocrit, CPDA-stored) reconstituted with 200mL of normal saline solution
Marik et al.[32]	23	Pulmonary sepsis, HIV, burns, perforated peptic ulcer, perforated gallbladder, diabetes, necrotizing fascitis, gangrenous appendix, pancreatitis, septicemia, liver failure, hepatitis, AAA, metastatic carcinoid, trauma, pancreatitis, flail chest	20.4	Hb < 10	750mL
Ronco et al.[40]	5	Acute respiratory failure secondary to AIDS related Pneumocystis carinni	ND	ND	600mL of packed RBC (70 percent hematocrit, CPDA-stored) reconstituted with 200mL of normal saline solution
Conrad et al.[41]	19	Septic shock	ND	Hb < 10	ND
Lorente et al.[42]	16	Severe sepsis	22.1	Hb < 10	800mL
Gramm et al.[43]	19	Multiple trauma or MSOF following other operations and sepsis	ND	ND	1,000mL (2U)
Mazza et al.[44]	29	Pneumonia, mesenteric infarction, urinary infection, pancreatitis, trauma, aortic aneurism, aortic iliac graft, total hip replacement, gastrectomy, appendicitis, colectomy, intestinal obstruction	12.5	Hb < 9	1 packed RBC unit in patients with Hb level between 8 and 9.0g/dL, 2 packed RBC units when they presented Hb level between 7 and 8g/dL, and 3 packed RBC units when the Hb level was lower than 7g/dL

APACHE - Acute Physiology and Chronic Health disease Classification System; RBC - red blood cell; ND - no data; HTA - arterial hypertension; CAD - coronary artery disease; CHF - congestive heart failure; DM - diabetes mellitus; CKD - chronic kidney disease; CPD – chronic pulmonary disease; CHC - chronic hepatic condition; CVD - cerebral vascular disease; SvO_2 - mixed venous oxygen saturation; BP - blood pressure; SVR - systemic vascular resistance; CPDA - citrate phosphate dextrose adenine; AAA - abdominal aortic aneurysm; MSOF - multiple system organ failure. *Subgroup of septic patients.

Table 4SA - General characteristics of the studies reporting microcirculatory parameters (near infrared spectroscopy, sidestream dark field/orthogonal polarization spectral)

Author	Journal	Institution	Study design	Time analyzed	Primary endpoints	Secondary endpoints
Sadaka et al.[11]	Annals of Intensive Care	ND	Prospective, observational study	ND	Sublingual microvascular perfusion and flow using SDF and on muscle tissue oxygenation, oxygen consumption, and microvascular reactivity using NIRS	Correlate the variables obtained from NIRS with those obtained from SDF. In-hospital mortality
Sakr et al.[12]	Critical Care of Medicine	Erasme Hospital	Prospective, observational study	ND	Hemodynamic and microvideoscopic assessments (OPS)	ND
Creteur et al.[13]	Critical Care	Erasme Hospital	Prospective, observational study	ND	Hemodynamic and NIRS derived variables	ND
Donati et al.[29]	Critical Care	AOU Ospedali Riuniti of Ancona	Prospective, randomized trials	Feb 2011 - 2012	SDF, StO_2, THI	ND
Damiani et al.[33]	PLoS One	AOU Ospedali Riuniti of Ancona	Secondary analysis of randomized trial	Feb 2011 - 2012	SDF, StO_2, THI	ND

ND - no data; SDF - sidestream dark field; NIRS - near infrared spectroscopy; OPS - orthogonal polarization spectral; AOU - Azienda Ospedaliera Universitaria; StO2 - tissular oxygen saturation; THI - tissue hemoglobin index.

Table 4SB - General characteristics of the studies reporting microcirculatory parameters (near infrared spectroscopy, sidestream dark field/orthogonal polarization spectral)

Author	Journal	Inclusion criteria	Exclusion criteria	Number of patients	Main pathology	Mean APACHE II at admission	Transfusion trigger	Number of RBC received (mean)
Sadaka et al.[11]	Annals of Intensive Care	Severe sepsis, clinically euvolemic (by CVP and/or echocardiogram) and in the first 12 hours of sepsis, with hemoglobin < 7.0, or for a hemoglobin between 7.0 and 9.0 with lactic acidosis, or central venous oxygen saturation < 70%	RBC transfusion in the preceding 72 hours, peripheral vascular disease, liver cirrhosis, age < 18 years, active bleeding, shock secondary to any other cause (cardiogenic, hemorrhagic, obstructive), and pregnancy	10	Severe sepsis	24	Hb < 7.0, or for a hemoglobin between 7.0 and 9.0 with lactic acidosis, or central venous oxygen saturation < 70%	ND
Sakr et al.[12]	Critical Care Medicine	Patients requiring transfusion, with severe sepsis, considered euvolemic, mechanically ventilated	Liver cirrhosis, RBC transfusion in the preceding 72 hours, shock owing to any other cause (cardiogenic, hemorrhagic, obstructive), oral injuries, rapid deterioration of hemodynamic status with the need to increase vasopressor dose in the 2 hours preceding transfusion, previous inclusion in the study	35	Severe sepsis and septic shock	35	Hb < 7 g/dL or between 7 and 9 g/dL in the presence of signs of altered tissue perfusion	1-2
Creteur et al.[13]	Critical Care	Patients hospitalized in the 31-bed Department of Intensive Care Medicine of Erasme Hospital with anemia requiring RBC transfusion	RBC transfusion in the preceding 72 hours, peripheral vascular disease, liver cirrhosis, age < 18 years, active bleeding, and pregnancy	14	Sepsis	15	Hb concentration either < 8g/dL or between 8 and 9g/dL in the presence of altered tissue perfusion (that is, elevated lactate levels) or coronary artery syndromes.	1
Donati et al.[29]	Critical Care	Sepsis, severe sepsis, or septic shock	< 18 years, previous blood transfusions during ICU stay, previous history of coagulation disorders, cardiogenic or hemorrhagic shock, pregnancy, and factors impeding the sublingual microcirculation evaluation	10	Sepsis, severe sepsis, or septic shock	ND	Hb levels of less than 8 g/dL or as indicated by the attending physician	2-3
Damiani et al.[33]	PLoS One	Sepsis, severe sepsis, or septic shock	< 18 years, previous blood transfusions during ICU stay, previous history of coagulation disorders, cardiogenic or hemorrhagic shock, pregnancy, and factors impeding the sublingual microcirculation evaluation	10	Sepsis, severe sepsis, or septic shock	ND	Hb levels of less than 8g/dL or as indicated by the attending physician	2-3

APACHE - Acute Physiology and Chronic Health disease Classification System; RBC - red blood cell; CVP - central venous pressure; Hb - hemoglobin; ND - no data; ICU - intensive care unit.

Table 5S - Level of evidence

Author	Publication year	Level of Evidence
Sadaka et al.[11]	2011	II
Sakr et al.[12]	2007	III
Creteur et al.[13]	2009	IV
Mazza et al.[14]	2015	II
Gilbert et al.[24]	1986	III
Silverman et al.[25]	1992	III
Sadaka et al.[26]	2014	II
Mark et al.[27]	2014	II
Fernandes et al.[28]	2001	II
Donati et al.[29]	2014	III
Steffes et al.[30]	1991	III
Ronco et al.[31]	1991	III
Marik et al.[32]	1993	III
Damiani et al.[33]	2015	III
Ronco et al.[40]	1990	IV
Conrad et al.[41]	1990	IV
Lorente et al.[42]	1993	IV
Gramm et al.[43]	1996	IV
Mazza et al.[44]	2005	IV

Evaluated according to the proposed evidence level of individual studies.

Table 6S - Studies reports about mean mixed venous oxygen saturation, oxygen extraction ratio, cardiac index, near infrared spectroscopy and sidestream dark field/ orthogonal polarization spectral before and after red blood cells transfusion

Author	Subgroup analysis (if available)	Total number of patients	Pre-transfusion measurements	Post transfusion measurements	p-value
Sadaka et al.[11]	NA	10	SvO_2 (mean, SD): 59.1 ± 9.2	SvO_2 (mean, SD): 63.8 ± 8.8	0.11
			StO_2% (mean, SD): 76.2 ± 9.3	StO_2% (mean, SD): 75.8 ± 8.1	0,8
		21	THI (mean, SD): 10.7 ± 3.4	THI (mean, SD): 12.2 ± 3.5	0,01
			Upslope StO_2 (mean, SD): 2.5±1.3	Upslope StO_2 (mean, SD): 2.6 ± 1.5	0,39
		11	Proportion of perfused small vessels (mean, SD): 37.6 ± 21.5	Proportion of perfused small vessels (mean, SD): 38.2 ± 21.8	0,85
			Perfused small vessel density (mean, SD): 9.5±4.8	Perfused small vessel density (mean, SD): 9.4 ± 4.8	0,91
Sakr et al.[12]	NA	35	SvO_2 (median, IQR): 64 (59 - 73)	SvO_2 (mean, SD): 67 (60 - 79)	> 0.05
			O_2ER (median, IQR): 33 (29 - 39)	O_2ER (mean, SD): 32 (20 - 38)	> 0.05
			CI (median, IQR): 3.6 (3 - 4.2)	CI (median, IQR): 3.7 (2.6 - 4.4)	> 0.05
Creteur et al.[13]	NA	14	SvO2 (median, IQR): 65 (51 - 72)	SvO_2 (median, IQR): 70 (52 - 74)	> 0.05
			O_2ER (median, IQR): 34 (28 - 48)	O_2ER (median, IQR): 28 (24 - 46)	> 0.05
			CI (median, IQR): 3 (2.7 - 3.4)	CI (median, IQR): 3.1 (2.9 - 3.4)	> 0.05
		18	StO_2% (median, IQR): 90 (81 - 94)	StO_2% (median, IQR): 90 (80 - 94)	> 0.05
			THI (median, IQR): 14 (13 - 17)	THI (median, IQR): 13 (11 - 18)	> 0.05
			Upslope StO_2 (median, IQR): 4.1 (2.1 - 5.4)	Upslope StO_2 (median, IQR): 3.8 (2.9 - 5.1)	> 0.05
			Downslope StO_2 (median, IQR): 22 (17 - 35)	Downslope StO_2 (median, IQR): 21 (16 - 32)	> 0.05
Mazza et al.[14]	Hb < 9 mg/dL	24	SvO2 (median, IQR): 72 (69 - 74)	SvO2 (median, IQR): 72 (71 - 73)	0.96
	Hb < 7 mg/dL	22	SvO2 (median, IQR): 68 (64 - 72)	SvO2 (median, IQR): 72 (69 - 75)	0.0001
Gilbert et al.[24]	Normal lactate	7	O_2ER (mean, SD): 29 ± 7	O_2ER (mean, SD): 24 ± 5	> 0.05
			CI (mean, SD): 3.6 ± 0.8	CI (mean, SD): 4 ± 0.8	> 0.05
	Elevated lactate	10	O_2ER (mean, SD): 31 ± 11	O_2ER (mean, SD): 26.3 ± 4	> 0.05
			CI (mean, SD): 3.9 ± 1.4	CI (mean, SD): 3.7 ± 1.4	> 0.05

Continue...

...continuation

Silverman et al.[25]	Normal gastric pHi	9	SvO2 (mean, SD): 72.4 ± 1.7	SvO2 (mean, SD): 73.5 ± 1.5	> 0.05
			CI (mean, SD): 5.4 ± 0.7	CI (mean, SD): 5.5 ± 0.7	> 0.05
	Low gastric pHi	10	SvO$_2$ (mean, SD): 70.6 ± 3.3	SvO$_2$ (mean, SD): 73.8 ± 2.7	< 0.05
			CI (mean, SD): 4.9±1.5	CI (mean, SD): 5.5 ± 1.7	> 0.05
Sadaka et al.[26]	1 RBC unit	46	SvO$_2$ (mean, SD): 63 ± 12	SvO$_2$ (mean, SD): 68 ± 10	0.02
	2 RBC units	16	SvO$_2$ (mean, SD): 63 ± 12	SvO$_2$ (mean, SD): 78 ± 11	0.01
Mark et al.[27]	NA	71	SvO$_2$ (median, IQR): 61 (55 - 68)	SvO$_2$ (median, IQR): 70 (60 - 75)	0.36
Fernandes et al.[28]	NA	10	CI (mean, SD): 4.7 ± 0.7	CI (mean, SD): 4.7 ± 0.1	> 0.05
Donati et al.[29]	Non leuko-depleted	10	StO$_2$% (median, IQR): 88 (80 - 90)	StO$_2$% (median, IQR): 90 (85 - 93)	0,03
			THI (median, IQR): 10.5 (7.8 - 11.2)	THI (median, IQR): 13.4 (10.4 - 15.8)	< 0,01
			Upslope StO$_2$ (median, IQR): 2.89 (1.35 - 3.67)	Upslope StO$_2$ (median, IQR): 3.2 (2.22 - 4.04)	0,01
			Downslope StO$_2$ (median, IQR): -9.5 (-11 - -8.5)	Downslope StO$_2$ (median, IQR): -9.5 (-11 - -8.5)	0.03
			Proportion of perfused small vessels (median, IQR): 88.5 (83.1 - 93.1)	Proportion of perfused small vessels (mean, SD): 90.6 (85.8 - 96.6)	0.32
			Perfused small vessel density (median, IQR): 16.2 (14.3 - 17.7)	Perfused small vessel density (mean, SD): 17.6 (13.5 - 21.4)	0.23
	Leuko-depleted	10	StO$_2$% (median, IQR): 83 (77 - 92)	StO$_2$% (median, IQR): 86 (82 - 99)	0.59
			THI (median, IQR): 10.3 (8.6 - 13.6)	THI (median, IQR): 13.8 (10.6 - 15.7)	0.02
			Upslope StO$_2$ (median, IQR): 3,30 (1.65 - 4.37)	Upslope StO$_2$ (median, IQR): 3.44 (2.18 - 4.86)	0.03
			Downslope StO$_2$ (median, IQR): -10,1 (-12.8 - -6.9)	Downslope StO$_2$ (median, IQR): -9.2 (-10.4 - -7.6)	0.56
			Proportion of perfused small vessels (median, IQR): 94.6 (82.9 - 95.9)	Proportion of perfused small vessels (median, IQR): 96.8 (94.7 - 98.9)	0.01
			Perfused small vessel density (median, IQR): 14,1 (11.8 - 17.2)	Perfused small vessel density (median, IQR): 18,8 (12.1 - 21.2)	0.04
Steffes et al.[30]	NA	21	SvO2 (mean, SD): 68 ± 7	SvO2 (mean, SD): 69 ± 7	> 0.05
			CI (mean, SD): 4.6 ± 1.3	CI (mean, SD): 4.7 ± 1.5	> 0.05
Ronco et al.[31]*	NA	12	SvO2 (mean): 68.8	SvO2 (mean): 71.2	ND
Marik et al.[32]	NA	23	O$_2$ER (mean, SD): 30.5 ± 8.1	O$_2$ER (mean, SD): 27.4 ± 7	< 0.05
			CI (mean, SD): 4.6 ± 1.7	CI (mean, SD): 4.4 ± 1.8	> 0.05
Damiani et al.[33]	Old Ricos	10	StO$_2$% (median, IQR): 82 (76 - 87)	StO$_2$% (median, IQR): 84 (75 - 93)	0.4
			THI (median, IQR): 11.4 (9.7 - 12.6)	THI (median, IQR): 13 (9.1 - 15.9)	0.05
			Upslope StO$_2$ (median, IQR): 2.65 (1.6 - 3.2)	Upslope StO$_2$ (median, IQR): 3.03 (2.31 - 3.7)	0.32
			Downslope StO$_2$ (median, IQR): -9.6 (-18.4 - -7.3)	Downslope StO$_2$ (median, IQR): -8.8 (-14.5 - -7.0)	0.16
			Proportion of perfused small vessels (median, IQR): 90,3 (86.3 - 96.3)	Proportion of perfused small vessels (median, IQR): 95.7 (93.3 - 97.1)	0.11
			Perfused small vessel density (median, IQR): 15.1 (11.8 - 17.6)	Perfused small vessel density (median, IQR): 16.5 (12.0 - 17.7)	0.23
Ronco et al.[40]	NA	5	SvO2 (mean, SD): 70 ± 4	SvO2 (mean, SD): 74 ± 4	ND
			O$_2$ER (mean, SD): 21.3 ± 2	O$_2$ER (mean, SD): 19 ± 2	< 0.26
			CI (mean, SD): 4.6 ± 1.2	CI (mean, SD): 4.6 ± 0.9	ND
Conrad et al.[41]	NA	19	SvO2 (mean, SD): 71 ± 2.3	SvO2 (mean, SD): 76 ± 1.6	< 0.01
			O$_2$ER (mean, SD): 26.3 ± 2.5	O$_2$ER (mean, SD): 21.3 ± 1.8	< 0.05
			CI (mean, SD): 4.6 ± 0.3	CI (mean, SD): 4.6 ± 0.3	> 0.05
Lorente et al.[42]	NA	16	SvO2 (mean, SD): 73.8 ± 1.7	SvO2 (mean, SD): 76.3 ± 1.6	0.001
			O$_2$ER (mean, SD): 21 ± 2	O$_2$ER(mean, SD): 19 ± 2	0.001
			CI (mean, SD): 3.62 ± 0.23	CI (mean, SD): 3.66 ± 0.25	> 0.05
Gramm et al.[43]	NA	19	O$_2$ER (mean, SD): 32 ± 2	O$_2$ER (mean, SD): 26 ± 1	0.001
			CI (mean, SD): 5.2 ± 0.4	CI (mean, SD): 5.0 ± 0.4	> 0.05
Mazza et al.[44]	NA	29	SvO2 (mean, SD): 64.3 ± 8.52	SvO2 (mean, SD): 67.4 ± 6.74	0.13

ND - no data; SvO$_2$ - mixed venous oxygen saturation; SD - standard deviations; StO$_2$ - tissue oxygen saturation; THI - tissue hemoglobin index; IQR - interquartile range; O$_2$ER - oxygen extraction ratio; CI - cardiac index; Hb - hemoglobin; RBC - red blood cells. *Subgroup of septic patients.

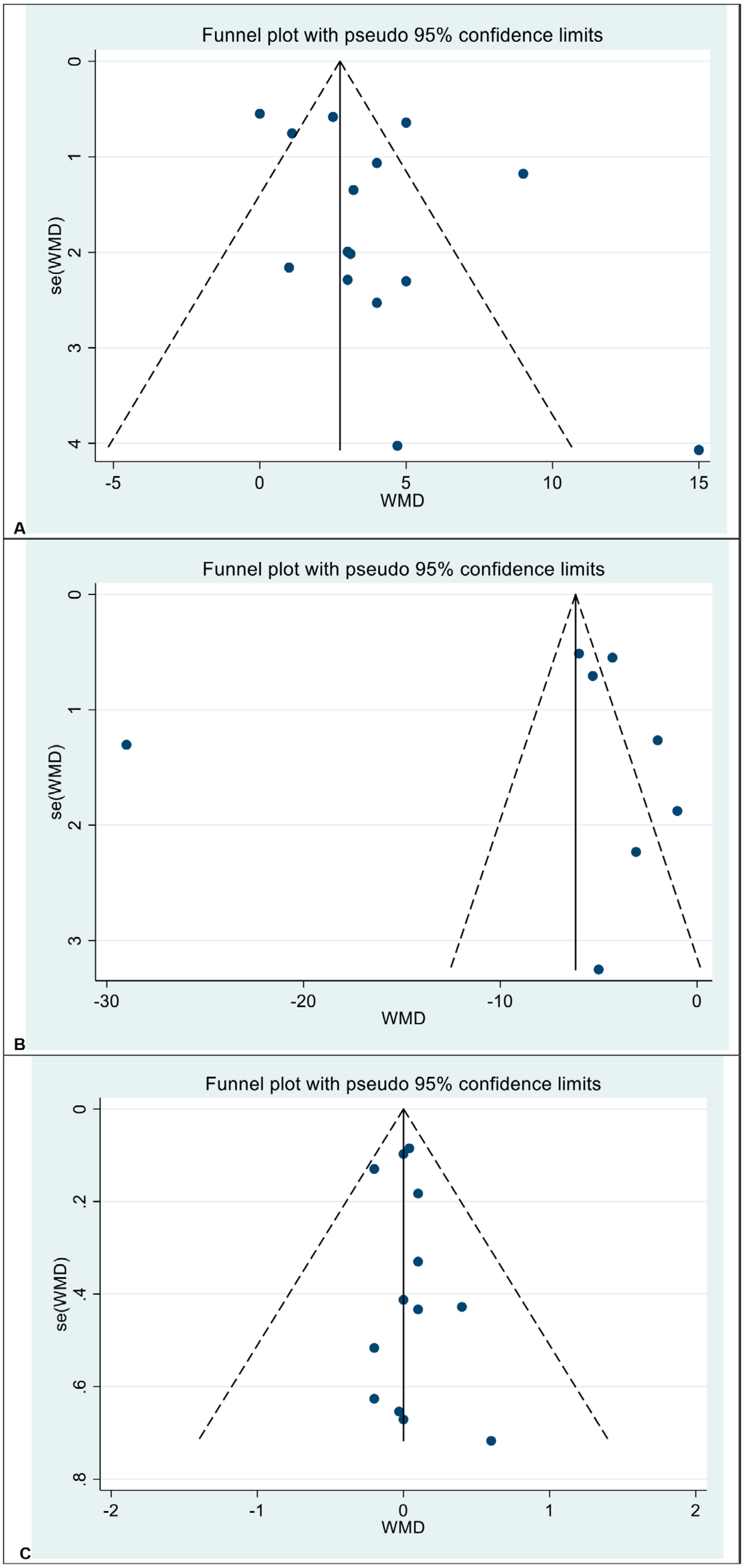

Figure 1S - Funnel plots with pseudo 95% confidence intervals limits for (A) mixed venous oxygen saturation, (B) oxygen extraction ratio and (C) cardiac index measurements.
All studies included.
WMD - weight mean difference.

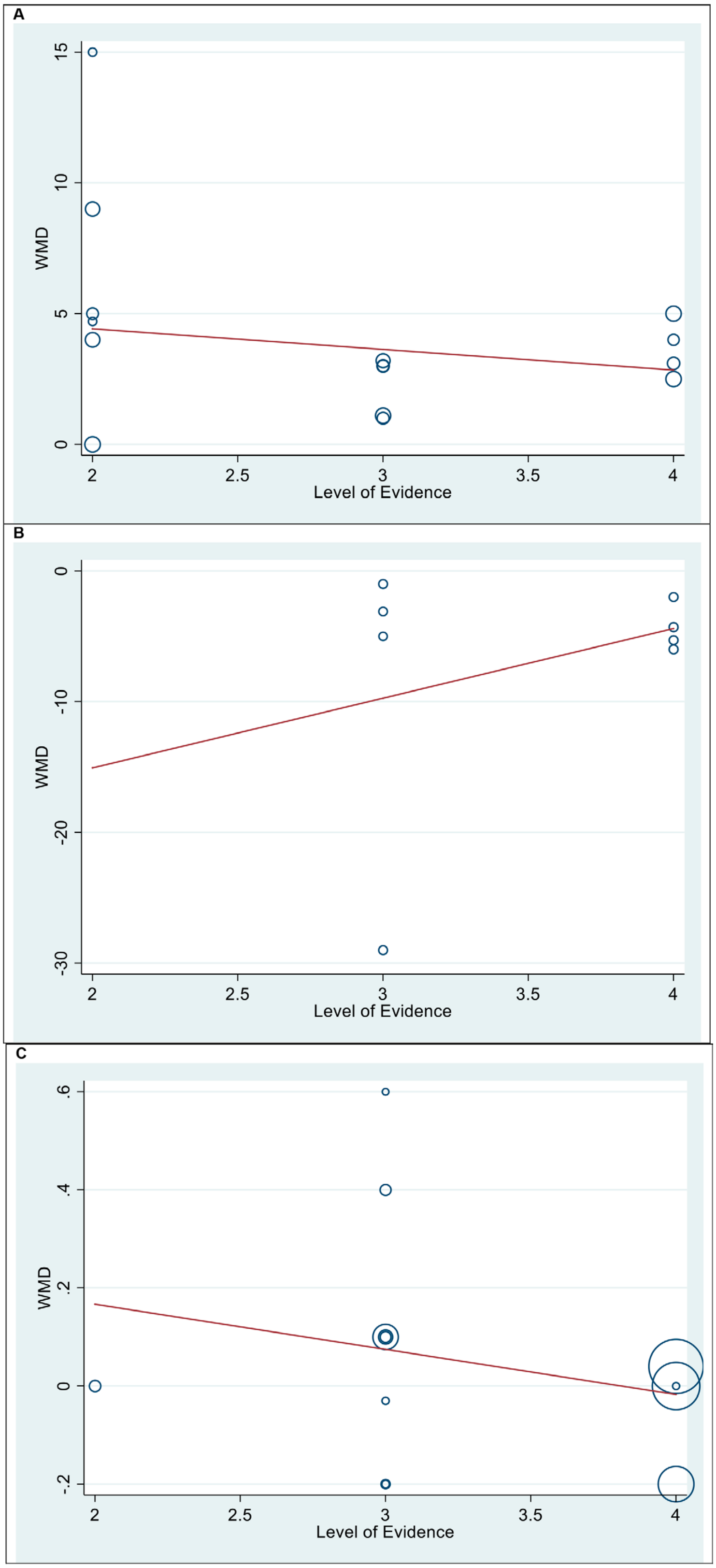

Figure 2S - Meta-regression for differences in the pooled mean differences of (A) mixed venous oxygen saturation, (B) oxygen extraction ratio and (C) cardiac index among levels of evidence.
All studies included.
WMD - weight mean difference.

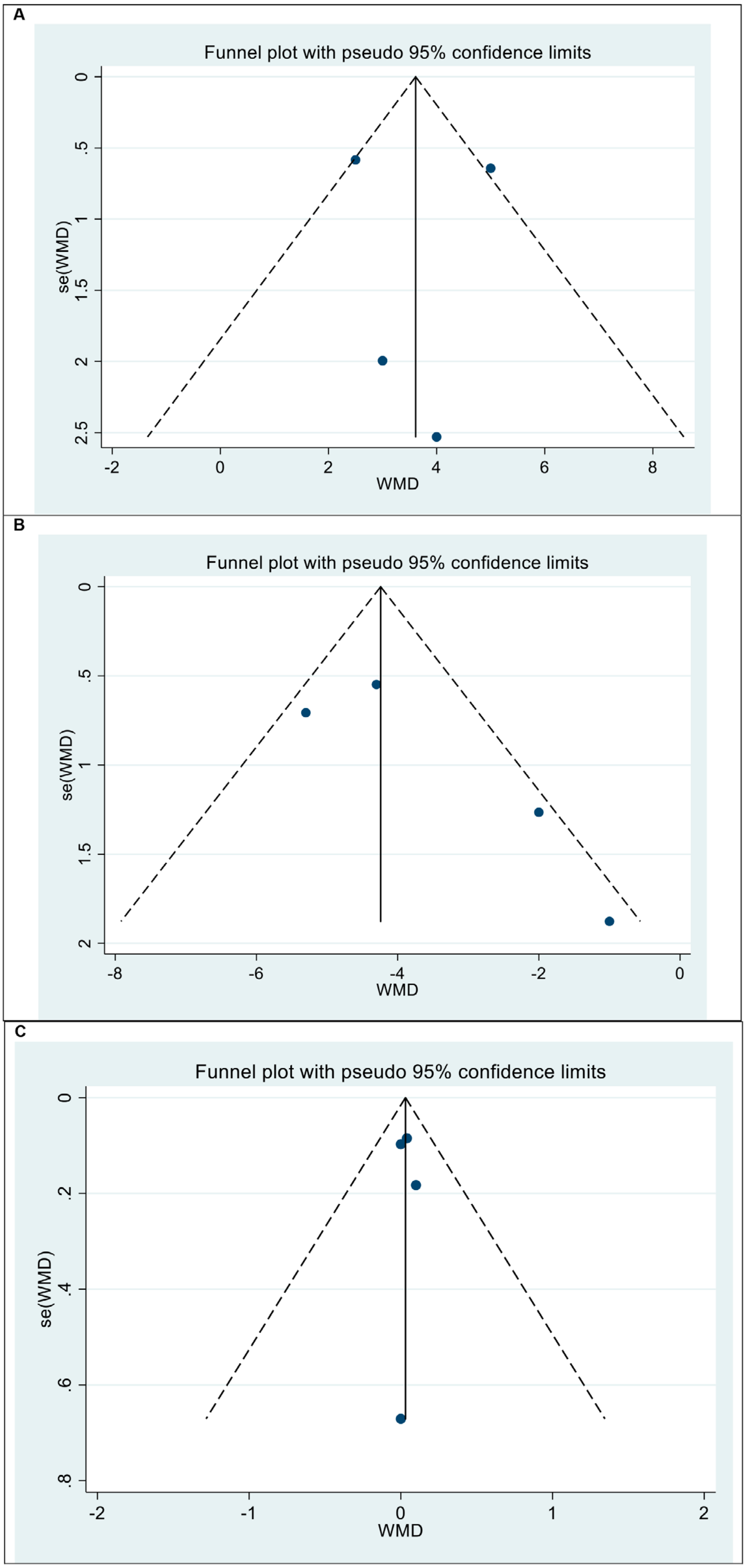

Figure 3S - Funnel plots with pseudo 95% confidence intervals limits for (A) mixed venous oxygen saturation, (B) oxygen extraction ratio and (C) cardiac index measurements.
Studies reporting simultaneously mixed venous oxygen saturation, oxygen extraction ratio and cardiac index included (n = 75).
WMD - weight mean difference.

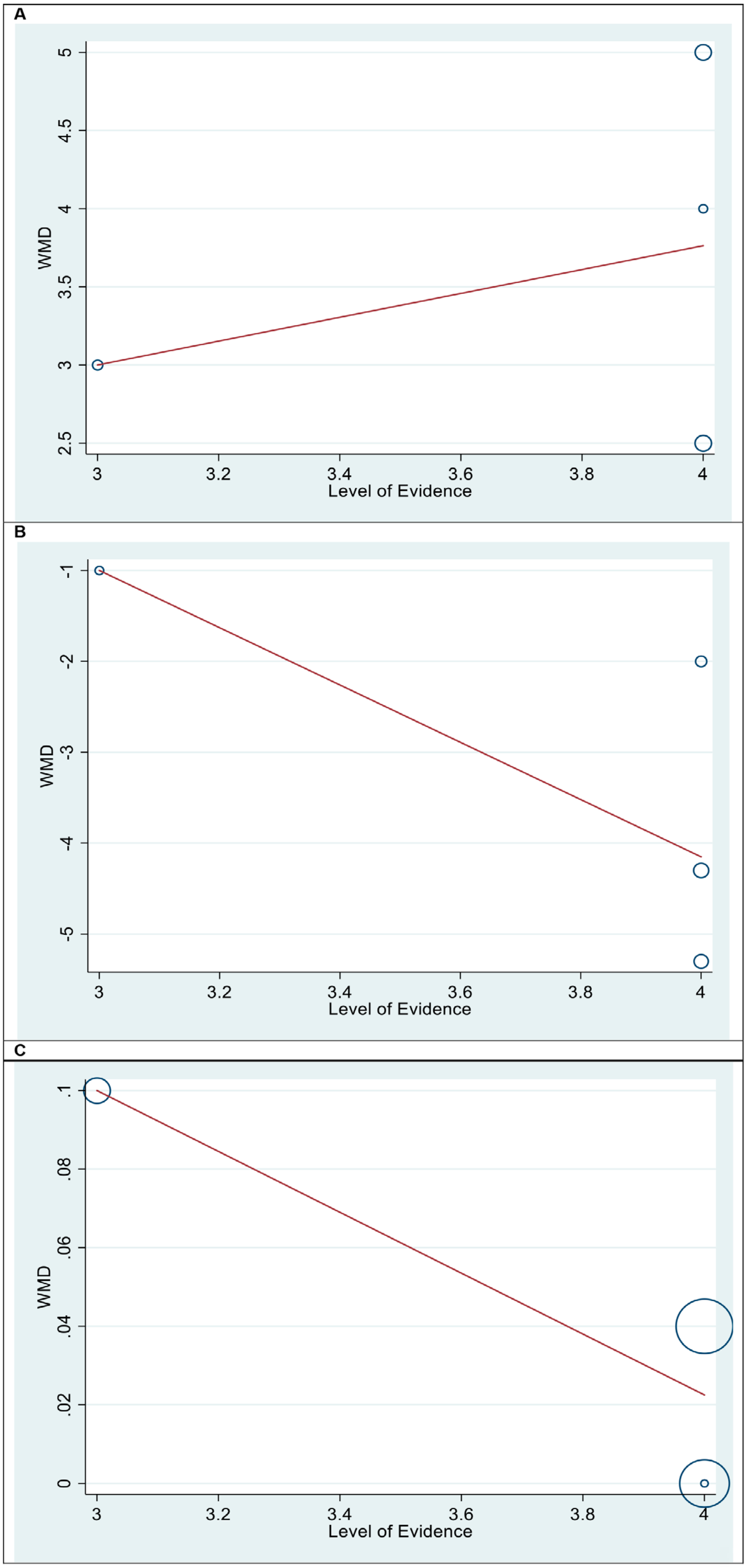

Figure 4S - Meta-regression for differences in the pooled mean differences of (A) mixed venous oxygen saturation, (B) oxygen extraction ratio and (C) cardiac index among levels of evidence of studies reporting simultaneously mixed venous oxygen saturation, oxygen extraction ratio and cardiac index (n = 75).
WMD - weight mean difference.
